# Addressing Criticisms of Large-Scale Marine Protected Areas

**DOI:** 10.1093/biosci/biy021

**Published:** 2018-04-05

**Authors:** Bethan C O’Leary, Natalie C Ban, Miriam Fernandez, Alan M Friedlander, Pablo García-Borboroglu, Yimnang Golbuu, Paolo Guidetti, Jean M Harris, Julie P Hawkins, Tim Langlois, Douglas J McCauley, Ellen K Pikitch, Robert H Richmond, Callum M Roberts

**Affiliations:** 1 Research associate at the Environment Department at the University of York, in the United Kingdom; 2 Associate professor at the School of Environmental Studies at the University of Victoria, in Canada; 3 Director at the Centro de Conservación Marina at Pontificia Universidad Católica de Chile, in Chile; 4 Chief scientist at the National Geographic Society's Pristine Seas Program and is affiliate faculty at the University of Hawai’i at Mānoa, in Honolulu; 5 Founder and president of the Global Penguin Society; a researcher at the National Research Council (CONICET), Argentina; and an affiliate professor at the University of Washington, in Seattle; 6 CEO at the Palau International Coral Reef Center; 7 Professor and director of the ECOMERS laboratory, CNRS & University of Nice Sophia Antipolis, part of the University Côte d’Azur, in France; 8 Leads the Scientific Services Division at the biodiversity conservation organization Ezemvelo KZN Wildlife, in South Africa; 9 Senior lecturer at the Environment Department at the University of York, in the United Kingdom; 10 Lecturer in the School of Biological Sciences and the Oceans Institute at the University of Western Australia; 11 Assistant professor at the Department of Ecology, Evolution, and Marine Biology and Marine Science Institute at the University of California Santa Barbara; 12 Executive Director of the Institute for Ocean Conservation Science and a Professor at the School of Marine and Atmospheric Sciences at Stony Brook University, USA; 13 Special Advisor to the President of Palau on Matters of Oceans and Seas; 14 Director and professor at the Kewalo Marine Laboratory at the University of Hawai’i at Mānoa, in Honolulu; 15 Professor at the Environment Department at the University of York, in the United Kingdom; 16 BO’L and CMR conceived the study; 17 BO’L, JPH, and CMR wrote the first draft; 18 All the authors reviewed and participated in revising the manuscript, including significantly contributing to the design of the manuscript and the interpretation of identified criticisms and responses. All authors approve of the final version of the manuscript. The authors declare no conflict of interest

**Keywords:** Convention on Biological Diversity conservation targets, marine protected areas, SDG 14, Sustainable Development Goal 14, very large marine protected areas

## Abstract

Designated large-scale marine protected areas (LSMPAs, 100,000 or more square kilometers) constitute over two-thirds of the approximately 6.6% of the ocean and approximately 14.5% of the exclusive economic zones within marine protected areas. Although LSMPAs have received support among scientists and conservation bodies for wilderness protection, regional ecological connectivity, and improving resilience to climate change, there are also concerns. We identified 10 common criticisms of LSMPAs along three themes: (1) placement, governance, and management; (2) political expediency; and (3) social–ecological value and cost. Through critical evaluation of scientific evidence, we discuss the value, achievements, challenges, and potential of LSMPAs in these arenas. We conclude that although some criticisms are valid and need addressing, none pertain exclusively to LSMPAs, and many involve challenges ubiquitous in management. We argue that LSMPAs are an important component of a diversified management portfolio that tempers potential losses, hedges against uncertainty, and enhances the probability of achieving sustainably managed oceans.

*Marine protected areas* (MPAs), places in the ocean where human activities are restricted to varying degrees, are often established with multiple objectives in mind. These include safeguarding biodiversity and ecosystem structure and function, enhancing fisheries by banking sufficient stocks to contribute to nearby populations or areas, helping support livelihoods and promoting more sustainable local economies, and preserving cultural values. To date (2018), there are approximately 13,000 MPAs worldwide, with a median size of approximately 2.5 square kilometers (km^2^; supplemental methods). Recently, however, there has been increasing interest in designating large-scale MPAs (LSMPAs; 100,000 km^2^ or larger; figure 1; Friedlander et al. 2016). These LSMPAs typically entail a range of protection levels, including multiple-use areas; “strongly” protected areas, where commercial activity is prohibited but recreational and subsistence fishing is allowed; and “fully” protected areas, where no extractive activities are permitted (table 1; figure 2; supplemental table S1; Lubchenco and Grorud-Colvert 2015). Although scientific evidence consistently shows that ecological benefits from MPAs (e.g., species or habitat recovery) are greatest for strongly or fully protected areas (Edgar et al. 2014), multiple-use MPAs can, in some contexts, also generate benefits (Di Franco et al. 2016) and may help balance social, ecological, and economic objectives (Day and Dobbs 2013).

**Figure 1.**
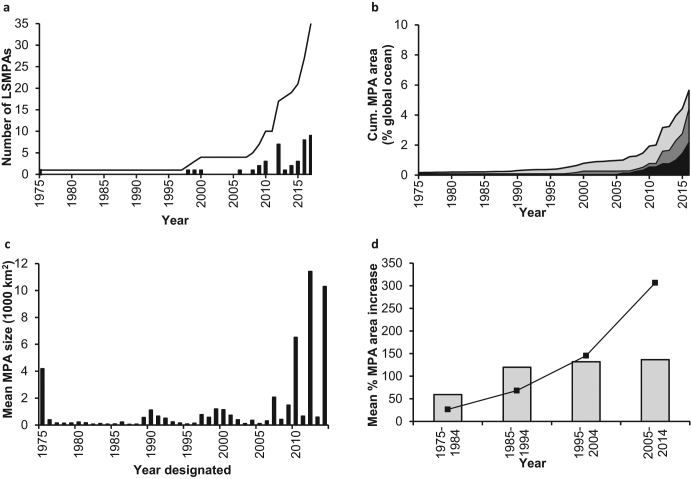
Global trends in marine protected area (MPA) coverage. (a) The number of large-scale MPAs (LSMPAs) designated or promised each year (black bars) and the cumulative number (black line) of LSMPAs designated or promised globally (1975–January 2018). No LSMPAs existed prior to 1975. (b) The cumulative percent coverage of all MPAs (light gray), all LSMPAs (dark gray), and strongly or fully protected area in LSMPAs (black) designated and promised globally (1975–2016). (c) The mean size of all MPAs designated each year (rather than a cumulative total, 1975–2014). The peaks correlate to years during which large areas were protected in LSMPAs. (d) The mean rate of increase (%) per decade in MPA coverage for all MPAs (black line) and for MPAs of 100,000 square kilometers or less (gray bars; 1975–2014). Note that the data from 2017 are not included in (b) because only eight LSMPAs were present in the data set. The data beyond 2014 are not included in (c) or (d) because of gaps in the WDPA database for small-scale MPAs. LSMPAs are detailed in supplemental table S1. The data for global MPA coverage were obtained from the IUCN-UNEP (2017) World Database on Protected Areas. Methods and further information are detailed in supplemental materials.

**Table 1.** A description of large-scale marine protected areas (LSMPAs) by legal status and protection regime.

	All LSMPAs	Fully or strongly protected LSMPAs	LSMPAs containing some fully or strongly protected area	Multiple-use LSMPAs				
Status	Number	Percentage of total area in LSMPAs	Number	Percentage of total area in LSMPAs	Number	Percentage of total area in LSMPAs	Number	Percentage of total area in LSMPAs
Designated	26	67.2	7	21.0	15	37.9	4	8.3
Promised	10	32.8	4	4.6	1	2.3	5	26.0

*Note:* “Percent of total area in LSMPAs” refers to the contribution the particular LSMPA category makes to the total area of LSMPAs around the world. For example, there are 26 legally designated LSMPAs in existence, which contribute 67.2% of the total area of LSMPAs globally; the remaining 32.8% is made up of LSMPAs promised by governments (i.e., plans for designation have been formally announced). Although we identified 35 LSMPAs in total (figure 2; supplemental table S1), 36 are detailed within this table because Rapa Nui Rahui (Easter Island) MPA is promised to expand on and replace the Motu Motiro Hiva Marine Park designated by Chile. The latter has therefore been counted as *designated* and the former as *promised*, with their respective areas (designated versus expansion area) used to calculate the percentage of total LSMPA area.

**Figure 2.**
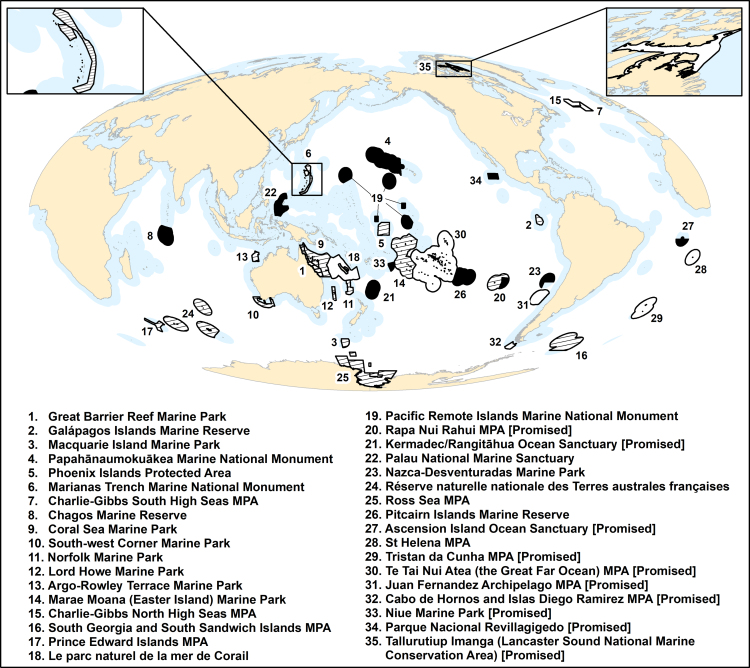
Global distribution of designated and promised LSMPAs as of January 2018. LSMPAs that are strongly or fully protected are shown in black, contain some strongly or fully protected area by stripes, and multiple-use MPAs by white with a black border. Promised LSMPAs are those identified by governments and are indicated in the legend. Note that the promised Rapa Nui Rahui (Easter Island) MPA (20) would encompass and replace the existing fully protected Motu Motiro Hiva Marine Park (shown as the black area within the Rapa Nui Rahui boundary). LSMPAs are detailed in supplemental table S1.

Founded in 1975 and covering approximately 344,000 km^2^, Australia's multiple-use Great Barrier Reef Marine Park was the world's only LSMPA for 23 years (figure 1a). By January 2018, however, governments had designated or promised (i.e., formally announced plans for designation) 35 LSMPAs (figures 1a and 2). Designated LSMPAs collectively constitute 70% of the approximately 6.6% of the ocean and 67% of the approximately 14.5% of exclusive economic zones (EEZ) within MPAs. Including promised LSMPAs increases global MPA coverage to approximately 8.9% of the ocean and approximately 19.8% of EEZs (figure 1b; supplemental methods). The recent expansion of LSMPAs is reflected by the jump in mean MPA size from 148 km^2^ for those designated in 1994 to 10,302 km^2^ in 2014 (figure 1c; supplemental methods), although ocean coverage by smaller MPAs continues to increase in step (figure 1c, 1d).

LSMPAs offer many advantages over their smaller counterparts. They encompass biologically connected and diverse ecosystems from coastal to pelagic and deep-sea regions, thereby benefiting both sedentary or sessile species and animals that can move or disperse large distances (e.g., White et al. 2017). They remove or limit direct anthropogenic stressors, which may promote greater ecological resilience to environmental disturbances and climate change and are more likely to encompass species’ range shifts under climate change (Roberts et al. 2017). They encourage “joined-up thinking” in management of large marine areas, and they offer rapid progress toward global MPA coverage targets (figure 1b, 1d), although greater efforts and resources to improve the effectiveness of MPAs around the world are still required (Gill et al. 2017). There is therefore considerable support for LSMPAs among marine scientists (e.g., Nelson and Bradner 2010, Graham NAJ and McClanahan 2013, Toonen et al. 2013, Singleton and Roberts 2014, Wilhelm et al. 2014, Friedlander et al. 2016).

LSMPAs have also, however, generated concerns (e.g., De Santo et al. 2011, De Santo 2013, Leenhardt et al. 2013, Devillers et al. 2015, Jones PJS and De Santo 2016, Hilborn 2017). We identified 10 criticisms of LSMPAs commonly aired in the scientific and popular literature along three themes: (1) placement, governance, and management; (2) political expediency; and (3) social–ecological value and cost (figure 3). Here, we evaluate these criticisms (grouped by theme) to constructively contribute to ongoing discussions on strategies for effective global marine management.

**Figure 3.**
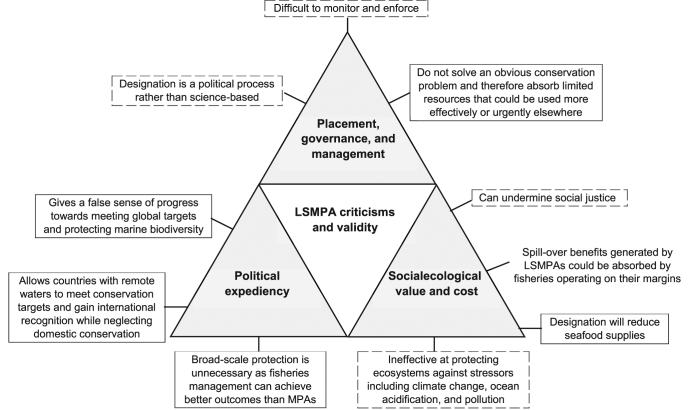
Ten commonly aired LSMPA criticisms categorized by theme and validity, according to the three conclusions of this article: (1) little available evidence showing that the criticism is valid (solid outline), (2) the criticism is valid but applies to MPAs of all scales (dashed outline), and (3) the criticism can also be seen as advantageous from a socioeconomic perspective and applies to fully or strongly protected MPAs of all scales (no outline).

## Theme 1: Placement, governance, and management

Three commonly aired criticisms relating to this theme were identified (figure 3). We evaluate the evidence for each in the sections below.

### Criticism: LSMPA designation is a political process rather than a science-based one (e.g., Leenhardt et al. 2013).

MPAs are one of several valuable tools for marine conservation and management. The decision to implement MPAs at whatever size is made by societal and political processes, and although political opportunity has featured strongly in the designation of many LSMPAs, most would not have been identified—or protection advocated for—without science highlighting their unique attributes and healthy conditions (e.g., Friedlander et al. 2014). Most LSMPAs presently occur in remote areas (figure 2) to help minimize biodiversity decline, particularly from future pressures; however, some, such as South Africa's Prince Edward Islands MPA, were created in response to existing fisheries threats (Lombard et al. 2007). The need for such action is supported by scientific evidence that shows ecosystems under fewer stressors have greater stability and resilience, may help contribute to climate change adaptation and mitigation, and ensure continuation of ecosystem services including fisheries (Roberts et al. 2017).

Political support is necessarily a major driver of MPAs, regardless of size (e.g., Fox et al. 2013). As remote areas usually contain relatively few direct stakeholders compared with heavily used areas, it is generally easier (both legally and with less opposition) and cheaper to establish marine protection in such places (McCrea-Strub et al. 2011, McCauley et al. 2013). Although remote areas may be viewed as politically expedient locations for protection, in places with high intensity and diversity of human use, LSMPAs are unlikely to be an appropriate tool, particularly if strongly or fully protected. This at least partly explains the tendency to locate LSMPAs away from such places. However, this propensity to place MPAs in areas with fewer human activities is not unique to LSMPAs. For example, in busier seascapes, marine spatial planning is often used to design networks of small-scale MPAs or zoning of multiple-use LSMPAs. This typically involves assigning a lower “cost” of protection to areas with fewer human uses to strategically locate MPAs in places that achieve conservation objectives while minimizing social and economic costs (Ban and Klein 2009). Although intensively used areas should not be ignored in conservation strategies, early protection of less affected areas is prudent.

### Criticism: LSMPAs do not solve an obvious conservation problem and therefore absorb limited resources that could be used more effectively or urgently elsewhere (e.g., Devillers et al. 2015).

There are several obvious conservation problems that LSMPAs are well placed to address, particularly where they are strongly or fully protected. Because overfishing and the associated collateral damage to habitat and ecosystem structure and function are pressing problems in most of the ocean, LSMPAs can help address these concerns and offer many advantages that fishery measures such as bycatch mitigation and single-species quotas do not confer (Graham N et al. 2007). Precautionary protection against emerging activities also offers proactive rather than reactive management, particularly for habitats such as the deep-sea or oligotrophic ecosystems (e.g., oceanic) that will likely take decades to millennia to recover from disturbance (e.g., Jones DOB et al. 2017).

LSMPAs have been criticized for being designated in remote waters “residual” to commercial interests and distant from the most serious threats, leading some to suggest that protection of these areas is not required and may divert attention from areas in more urgent need (e.g., Devillers et al. 2015, Agardy et al. 2016). A related criticism is that LSMPAs protect regions of low conservation value given their large component of less diverse pelagic and deep seafloor habitat compared with more diverse and productive coastal habitats. However, although some LSMPAs may currently experience limited direct human impacts (supplemental figure S2), threats remain (Friedlander et al. 2014, Davies et al. 2017), and history shows that with increasing human population and resource demand, no unused area can be presumed to remain undisturbed in perpetuity (Halpern et al. 2015). Proactive protection of ocean “wilderness” areas against future exploitation could offer large long-term benefits to marine biodiversity and ecosystem services (Graham NAJ and McClanahan 2013, D’agata et al. 2016, Roberts et al. 2017). On land, almost one-tenth of wilderness areas have been lost over the past two decades, greatly exceeding the rate at which new terrestrial protected areas have been created (Watson et al. 2016). In the sea, LSMPAs therefore offer a time-limited opportunity to protect sites before they experience increased commercial interest and impacts; that is, there is a premium on early designation.

Small MPAs can offer important local benefits (Di Franco et al. 2016, Giakoumi et al. 2017); however, given the wide spatial scale of interconnected marine ecosystems, with characteristically indistinct boundaries, effective management needs to be large scale and encompass the water column used by pelagic species. This way, it can better accommodate ecological processes and benefit highly mobile taxa such as whales, sharks, turtles, seabirds, and straddling fish stocks well known for their vulnerability to human pressures (e.g., Sumaila et al. 2015). Marine spatial planning goes some way toward achieving this in places where LSMPAs are not appropriate; however, the larger areas protected by LSMPAs not only better reflect the ranges of such species but also can serve as protected corridors of connectivity among habitats in ways not afforded by smaller MPAs. Although smaller MPAs can benefit species that move across their boundaries (e.g., Claudet et al. 2010), protection through LSMPAs could strengthen these outcomes (Edgar et al. 2014, Sumaila et al. 2015). Although even the largest LSMPAs will not offer complete protection to the oceans’ most mobile species, emerging evidence suggests these species may benefit from spatial protection nonetheless (Mee et al. 2017, White et al. 2017). Complementary management in areas surrounding LSMPAs will, however, remain essential for sustainable ocean management.

Resources for environmental protection are limited and must be invested wisely. Larger MPAs offer economies of scale being less expensive to manage per unit area than smaller MPAs (Balmford et al. 2004). Protecting areas before degradation occurs also helps insure against needing costly restoration measures. With simple management schemes (e.g., strongly or fully protected), LSMPAs may also be proportionally cheaper to implement and manage than multiple small networked MPAs or MPAs with complex zoning (Balmford et al. 2004, Ban et al. 2011, McCrea-Strub et al. 2011). Although designating LSMPAs will require large capital investment and incur ongoing costs, this is also true of smaller MPAs (Gill et al. 2017).

### Criticism: LSMPAs are difficult to monitor and enforce (e.g., De Santo 2013).

Because LSMPAs are mainly remote and in sparsely populated or uninhabited areas (figure 2), they present limited opportunities for comanagement and therefore require innovative mechanisms to ensure compliance and monitor responses to protection. Numerous rapidly emerging technologies (e.g., drone, radar, and satellite observation) and their decreasing costs offer opportunities for cost-effective monitoring of marine life and human activities. For example, advances in satellite tracking of vessels have enabled remote monitoring and detection of illegal fishing (McCauley et al. 2016) and near-real-time surveillance of fishing activities (e.g., see Global Fishing Watch, *http://globalfishingwatch.org*). This information can be used to provide evidence of illegal activity and direct enforcement vessels to those suspected of engaging in illegal activities, streamlining patrol activities. For instance, in 2015, it assisted with the interdiction of a boat suspected of illegally fishing within Kiribati's Phoenix Islands Protected Area (McCauley et al. 2016). Improvements in satellite imaging, remote sensing, and drone technologies, together with greater cooperation promoted by the FAO’s Port State Measures Agreement (www.fao.org/fishery/psm/agreement/en), which came into effect in 2016, will make LSMPA monitoring and enforcement increasingly effective and affordable, although by their nature, enforcement actions will remain costlier than monitoring. Although enforcement of LSMPAs remains challenging, this does not invalidate LSMPA designation. Moreover, continued involvement and partnerships with stakeholders will also improve compliance, which is always preferable and more cost-effective than enforcement actions alone.

This criticism assumes that monitoring and enforcing MPAs in more populated areas is easier. However, places with severe pressures on resources require costly and intensive monitoring and enforcement. MPAs in such settings have regularly been criticized for inadequacy in this respect, and ensuring adequate levels of protection, as well as staff and budget capacity for management, is essential for effective protection (Edgar et al. 2014, Gill et al. 2017). Recognizing this, financial commitments for protection are increasingly being made by nonprofit organizations and national governments. For example, at the 2016 Our Ocean conference, nonprofit organizations pledged US$48 million to the Wildlife Conservation Society MPA Fund that aims to expand and improve MPA effectiveness. Concurrently, the UK government committed approximately US$21 million over 4 years to support the implementation, management, surveillance, and enforcement of LSMPAs in its overseas territories.

## Theme 2: Political expediency

Three commonly aired criticisms relating to this theme were identified (figure 3). We evaluate the evidence for each in the sections below.

### Criticism: LSMPA designation gives a false sense of progress toward meeting global targets and protecting marine biodiversity (e.g., Jones PJS and De Santo 2016).

The Convention on Biological Diversity Aichi target 11 (also adopted as Sustainable Development Goal 14.5) commits signatory governments to protect 10% or more of marine environments by 2020 in “effectively and equitably managed, ecologically representative and well-connected” area-based conservation measures (CBD 2010). Undoubtedly, LSMPA designations have contributed substantially to meeting global targets for MPA coverage (figure 1); however, they are also helping meet the other elements of this target, and these contributions will increase as they become better managed.

**Figure 4.**
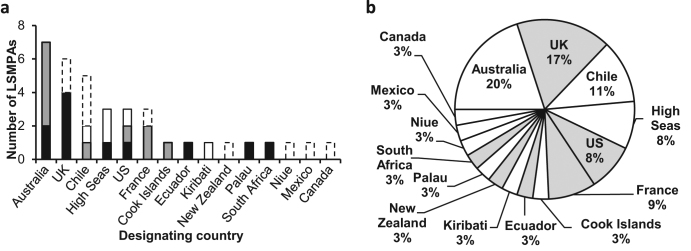
Details of countries designating large-scale marine protected areas (LSMPAs), available management, and jurisdictional location. (a) The number of LSMPAs designated and promised by country where there is an identifiable management plan in force (black) or in preparation (dark gray), where no management plan could be identified (white), and where management is not applicable because the MPA is not yet legally designated (white with dashed border). (b) A breakdown of countries that have designated or promised LSMPAs (n = 35). The shaded segments indicate where all LSMPAs of that country are in remote waters (overseas territories or distant waters). LSMPAs are detailed in supplemental table S1. Note that Chile has five LSMPAs detailed in (a) to accurately reflect management differences between the designated Motu Motiro Hiva Marine Park and the promised Rapa Nui Rahui (Easter Island) MPA, which, if designated, would replace the former. To accurately represent Chile in terms of contribution to global numbers of LSMPAs, only four LSMPAs are included in (b).

The designation of MPAs often follows a sequential process: creation, development of a management plan, then implementation of management (including monitoring and enforcement) activities. Although it may be preferable to complete this process simultaneously, often, the initial designation is necessary to provide the political impetus, public expectation, and even funding for the latter two steps (Giakoumi et al. 2017). Of the 26 designated sites, we identified 11 LSMPAs with management plans in force, 10 with plans in preparation, and 5 with no identifiable management (figure 4a; supplemental table S1). The presence of management plans (existing or in preparation) for the majority of LSMPAs counters potential “paper park” claims, although the effectiveness of management plans depends on monitoring and enforcement action and capacity and this requires further work to identify. For the five sites without clear management, it is imperative that management plans be implemented and/or made publicly available to ensure LSMPAs contribute meaningfully to the emerging global MPA network. However, although some LSMPAs do not yet have formal management plans, this does not mean they will be ineffective once plans are in place. Moreover, a more prudent response to ineffective management is to improve on existing MPAs rather than abolishing them or preventing additional ones from being established.

With societal support essential for management effectiveness, equitability is a crucial component of MPA design, although its incorporation is challenged by different understandings of what equity means and how to achieve it (Klein et al. 2015). LSMPAs offer an opportunity to contribute to local and global equitability through their objectives and design, which we discuss later. Given their size and holistic approach to biodiversity protection, LSMPAs also offer greater opportunities than networks of smaller MPAs for regional ecological representation and connectivity because they can capture a variety of fixed and ephemeral habitats.

It is true that LSMPAs are presently not fully ecologically representative or well connected at the global scale. Nor, on their own, are they likely to provide enough protection to highly mobile species, although they could offer substantial benefits to more resident elements of populations (e.g., White et al. 2017). Even if considered with all existing smaller MPAs, these broader goals would still not be met because of our current inability to protect and represent species and habitats in areas beyond national jurisdiction, including the deep-sea and pelagic realms. However, we are only at the start of the process for developing effective large-scale ocean management, and it takes time to enact ambition. In addition, although LSMPAs offer rapid progress toward meeting global targets, a fully representative global MPA network cannot be constructed by protecting only 10% of the seas. To reach goals of ecological representation and connectivity, it will be necessary to increase coverage ambition and extend protection beyond EEZs (O’Leary et al. 2016). Moreover, the 10% coverage target does not differentiate between the level of protection afforded to different MPAs despite this being a key driver of ecological benefits (Edgar et al. 2014). Expanding the coverage of strongly or fully protected MPAs across all seascapes must therefore also be an aspiration. Finally, strong fisheries management outside of protected areas is required to support LSMPAs, and likewise, well-managed LSMPAs can help reinforce the goals of managers in fishing zones (Ban et al. 2017).

### Criticism: LSMPAs allow countries with remote waters to meet conservation targets and gain international recognition while neglecting domestic conservation (e.g., Jones PJS and De Santo 2016).

The crux of this criticism is that conservation close to population centers suffers because of protection given to remote areas. However, eight nations, including small island developing states, have made or are in the process of making critically important contributions to local domestic conservation via the establishment of ambitious LSMPAs (figure 4b). Nations such as Palau and Kiribati, for example, have established LSMPAs in their coastal waters that are approximately 500 and 1000 times larger, respectively, than their own land masses. Although 17 of the 35 LSMPAs we identified are in the remote waters of seven countries (table 2; figure 4b), marine conservation efforts began in their local domestic waters and are ongoing there (table 2). LSMPAs are complementary to those efforts, not substitutes. It is often the case, however, that MPAs in local domestic waters are subject to much weaker protection than those in distant waters. For example, the United Kingdom currently has strongly or fully protected approximately 1,495,000 km^2^ in its overseas territories but only 7.5 km^2^ (less than 0.001%) within the EEZ of the British Isles. Likewise, the United States has less than 1% of seas in continental US waters under strong or full protection compared with approximately 43% (approximately 2.6 million km^2^) of remote waters. Similarly, Chile has protected less than 1% of their mainland EEZ in strongly or fully protected MPAs versus 27% (approximately 450,000 km^2^) of their EEZ around remote oceanic islands. Given the contingency of beneficial outcomes on high-level protection (Edgar et al. 2014), local domestic waters of these countries clearly need much stronger protection. Nonetheless, lessons regarding MPA effectiveness must also be applied to LSMPAs. Currently, 47.9% of the area within designated LSMPAs (7,991,520 km^2^ out of 16,670,988 km^2^) is strongly or fully protected (table S1). However, this falls to 19.8% for promised LSMPAs (1,609,641 km^2^ out of 8,137,596 km^2^; table S1). To ensure that LSMPAs deliver anticipated benefits, adequate levels of protection will be key.

**Table 2.** The number of large-scale marine protected areas (LSMPAs) designated in remote waters by the designating country together with details of local domestic MPAs.

Designating Country	Number of remote LSMPAs/total number of LSMPAs	Details of local domestic MPAs
UK	6/6	As of December 2017, the United Kingdom has 293 MPAs covering approximately 23% of national waters (JNCC 2017). In recent years, extensive stakeholder consultations for new MPAs were undertaken, leading to approximately 80 being designated between 2013 and 2016, with a further 50 sites proposed in November 2016 and currently under consideration (JNCC 2017).
US	3/3	The United States has designated more than 1,700 MPAs and began to formally develop a ‘National System of MPAs’ in 2000 (National MPA Center 2015). Efforts to expand the national system and improve MPA management effectiveness and capacity are ongoing (National MPA Center 2015).
France	3/3	In 2006, France created the Marine Protected Areas Agency (integrated in 2017 into the French Agency for Biodiversity), and by 2015, 26.3% of local domestic waters were covered by MPAs (MEDDE 2015). There are currently plans to extend the existing network, improve management, and increase the area fully protected (MEDDE 2015).
Chile	2/4	Chile has designated 24 MPAs since 1997, with protection ranging from multiple use to fully protected. In recent years, there have been efforts to increase the number of MPAs and to improve management effectiveness (Gelcich et al. 2015).
Ecuador	1/1	Ecuador established its first continental coastline MPAs in 1979, and in recent years, the number of MPAs has increased substantially with efforts to encourage comanagement and citizen participation to improve governance ongoing (Gravez et al. 2013).
New Zealand	1/1	New Zealand was a pioneer of highly protected marine reserves, and 44 of these plus several MPAs now exist in their territorial waters (12 or fewer nautical miles; DEC 2017). No MPAs currently exist in the rest of New Zealand's EEZ, although area-based restrictions are in place for fisheries. Proposals released in early 2016 to reform MPA creation are also confined to territorial seas.
South Africa	1/1	South Africa currently has 24 domestic MPAs designated covering approximately 4,724 km^2^ (DEA 2017); in early 2016, a further 22 MPAs covering 70,000 km^2^ were proposed but are yet to be adopted.

*Note:* Details of all LSMPAs are provided in supplemental table S1.

### Criticism: Broadscale protection via LSMPAs is unnecessary as fisheries management can achieve better outcomes (e.g., Hilborn 2016).

Effective fisheries management can achieve several of the benefits that well-managed MPAs offer; for example, reducing fishing effort can rebuild overexploited fish stocks (Costello et al. 2016). However, fisheries management focuses on species of commercial interest with nontarget or no longer commercially viable resources being of lesser consideration, and it often fails to account for the collateral impacts of fishing (Travis et al. 2014). Measures such as bycatch mitigation, gear restrictions, and seasonal closures may reduce some of the broader ecosystem impacts of fisheries (Graham N et al. 2007). However, MPAs embody long-term ecosystem-based management, protecting vulnerable and under- and unvalued species and helping secure ecosystem integrity through maintenance of trophic linkages, things that usually go beyond the mandate or competence of fishery managers (Travis et al. 2014).

LSMPAs will also help to insure against uncertainty in fisheries management and expansion of human activities, encompass a scale approaching that necessary for protection of highly mobile species, and extend population age structures, enabling greater numbers of large individuals critical to population replenishment to build up. Undoubtedly, however, broadscale and effective fishery management is an essential complement to LSMPAs. Finally, this criticism assumes objectives of MPAs focus solely on addressing fishery problems, whereas MPAs are established to achieve a diverse range of objectives outside the remit of fishery management, including addressing threats from other activities, such as maritime traffic or oil and mineral exploration and exploitation.

## Theme 3: Social–ecological value and cost

Four commonly aired criticisms relating to this theme were identified (figure 3). We evaluate the evidence for each in the sections below.

### Criticism: LSMPAs can undermine social justice (e.g., De Santo et al. 2011).

Concerns about the relationship between LSMPAs and social justice must be taken very seriously given the critical need to balance and harmonize goals for biodiversity protection with human rights. Complaints about infringement of social justice are usually made where LSMPAs have been designated by top-down processes. Concerns over political motivations (e.g., Chagos Marine Reserve; De Santo et al. 2011), lack of universal support (e.g., Easter Island Marine Park; Radwin 2016), and inadequate stakeholder consultation (e.g., New Zealand's Kermadec Ocean Sanctuary; Newman 2016) have all been raised. In the case of the Chagos Marine Reserve, for example, a leaked confidential UK government memo in 2010 suggested prevention of resettlement of the archipelago by Chagossians was a motivation for the LSMPA. However, designation was under the proviso that “should circumstances change, all the options for a marine protected area may need to be reconsidered” (FCO 2009), leaving the opportunity open for island resettlement. Recent legal rulings state that the LSMPA has no bearing on the UK government's decision not to permit resettlement (UK Supreme Court 2016), whatever private communications were had. Thus, although the forced expulsion of the Chagossians in the 1960s and 1970s is undoubtedly a case of undermined social justice, the claim that the establishment of the Chagos Marine Reserve furthers this is, in our view, inaccurate.

It is essential that people are considered alongside nature in any marine management decision in order to not only promote equity and justice but also improve compliance, thereby reducing enforcement costs and increasing management effectiveness (Agardy et al. 2016, Di Franco et al. 2016). For example, involvement of Native Hawaiians led to cultural heritage and the importance of an uninterrupted seascape for traditional navigation featuring prominently in the Papahānaumokuākea Marine National Monument designation (Friedlander et al. 2016). The 2016 expansion was initiated by a group of Native Hawaiians with full support from the Office of Hawaiian Affairs, and the reserve has been designed to exclude commercial fishing but allow Native Hawaiians to exploit resources for subsistence and cultural purposes (Kikiloi et al. 2017). Similarly, the Pitcairn Islands Marine Reserve covers the entirety of the EEZ, excluding halos around islands and an offshore reef so that the local population can continue fishing for subsistence and trade. Likewise, the Palau National Marine Sanctuary extends from 20 nautical miles beyond coastlines to Palau's EEZ boundary (equivalent to 80% of the EEZ), with management of 0–20 nautical miles devolved and exclusive access given to local communities in accordance with land-based boundaries. More recently, continued engagement with the Rapa Nui has led to majority support for the designation of the Rapa Nui Rahui (Easter Island) MPA.

More generally, stakeholder involvement in the design of LSMPA management zones also contributes to equitable management (e.g., Great Barrier Reef Marine Park; Day and Dobbs 2013). In reality, however, there is almost never universal support for any change in management, and it is unrealistic to expect this for all MPAs, regardless of size, although where local communities are small it is possible (e.g., Pitcairn Islands Marine Reserve; Pitcairn Islands Tourism 2017).

Many of the social concerns raised regarding LSMPA creation relate to potential future use of an area rather than displacement of existing activities, and focus on consumptive rather than nonconsumptive users. However, LSMPAs have generally been located in areas subject to fewer commercial interests, particularly in terms of commercial fisheries activity (figure S2). The low overlap with commercial fisheries, combined with the design of LSMPAs to permit traditional and artisanal fishing by local communities, suggests displacement of fishers is not currently a major concern for the majority of LSMPAs. Where local people are present, it is essential that LSMPAs are designated with their involvement and support. However, if they are absent, then top-down political designation may be a more valid option, so long as appropriate consideration of an area's existing uses and values occurs (Gruby et al. 2016, Ban et al. 2017, Bennett et al. 2017, Christie et al. 2017).

At a global scale, current ocean management has, in some cases, led to situations of social injustice in which the loss of biodiversity and its associated benefits has occurred by capitalist forces at the expense of those less empowered, leading to unequal distribution of benefits at the cost of shared resources. For example, 10 countries capture 71% of the landed value of high-seas fish stocks, an area where LSMPAs could help reduce inequality in the distribution of fisheries benefits among the world's nations in a cost-effective fashion (Sumaila et al. 2015, Cheung et al. 2017). Similarly, LSMPAs zoned to allow some extractive use for local people could also be used to promote social justice and food security by enhancing local catches from spillover effects and helping to combat unsustainable fishing by industrial fleets (Standing 2015). For example, the Palau National Marine Sanctuary has been designed to redistribute benefits to Palauans by restricting access by foreign fishing fleets and promoting local management.

Ocean resources are shared and of wider importance than simply direct economic value. LSMPAs are therefore well suited to address social justice and equity, including intergenerational equity, provided they are appropriately placed and consulted on (Ban et al. 2017, Bennett et al. 2017, Christie et al. 2017). Finally, the benefits arising from protection and improved ocean management will be experienced globally and include ecosystem services of wide public interest, such as climate regulation and biodiversity refugia.

### ***Criticism: Spillover benefits generated by LSMPAs could be absorbed by fishers operating on their margins (*** ***Agardy 2017*** ***)***.

The phenomenon of “fishing-the-line” around MPAs is well documented and enables fishers to benefit from the spillover, or net emigration, of commercially valuable species (Di Lorenzo et al. 2016). Such a response is rational: They can fish more effectively at less expense through reduced fuel and time spent searching for fish due to greater stock densities. Furthermore, fishing-the-line provides evidence that an MPA is appropriately located for the target fish species and indicates management effectiveness.

Fishing-the-line may be perceived as disadvantageous to a nation that has established a LSMPA because many, but not all, extend to EEZ boundaries. Any spillover benefits at their outer boundaries will therefore accrue beyond national jurisdiction. This could be remedied by making the outer strongly or fully protected limits within national waters or by zoning the protected area to allow domestic fleets to benefit. In the Galapagos Marine Reserve, for example, the tuna purse-seine fleet (66 vessels from 10 nationalities) can be seen fishing-the-line around the reserve (Boerder et al. 2017), suggesting that Ecuador is benefiting financially through granting access.

Although fishing-the-line may limit the geographic spread of spillover, it should not affect the overall amount unless fishers catch more fish than can be biologically sustained or concentrate in particularly sensitive areas. Nor will it affect the export of eggs and larvae to surrounding areas, which is one of the major and spatially most far-reaching benefits of protection (Di Lorenzo et al. 2016). Fishing-the-line also offers the opportunity to encourage stakeholder support for protective measures, although in overexploited fisheries, reductions in total fishing effort will likely be required to ensure sustainability (Kellner et al. 2007).

### Criticism: LSMPA designation will reduce seafood supplies (e.g., Hilborn 2017).

Global food security, particularly of lower-income countries, is already threatened by overexploitation of fish stocks, and this is likely to worsen under climate change (Golden et al. 2016). The fishery benefits of LSMPAs are an emerging field of study. However, in areas with high fishing effort, LSMPAs may already be providing localized fishery benefits (Ban et al. 2017). For example, the western and southwestern boundary of the Galapagos Marine Reserve attracts greater tuna fishing effort and supports a higher catch per unit effort than the remainder of Ecuador's EEZ, although declines in catch per unit effort are occurring throughout Ecuador's waters (Boerder et al. 2017). Other LSMPAs, such as the Palau National Marine Sanctuary, have been designed to enhance local seafood supplies. Spatial protection has long been a tool in fisheries management, and LSMPAs are no exception. Given that effective protection of important areas, such as spawning or nursery grounds, combined with good fishery management can help rebuild exploited fish stocks, LSMPAs may, in fact, contribute to increasing seafood supply while simultaneously achieving many other benefits, such as habitat protection and climate resilience. (Sumaila et al. 2015, Cheung et al. 2017).

### Criticism: LSMPAs are ineffective at protecting ecosystems against stressors such as climate change, ocean acidification, and pollution (e.g., Hilborn 2017).

This same criticism can also be applied to good fisheries management, with which MPAs are often compared. No one tool can achieve all goals for ocean management, and LSMPAs will not be able to protect against all anthropogenic impacts, particularly those that diffuse across boundaries. That said, LSMPAs are likely to help promote ecosystem resilience and adaptation potential to changing environmental conditions. For example, LSMPAs may encompass range shifts of marine species under climate change, reduce cumulative stressors on ecosystems enabling faster recovery from climatic impacts, promote larger populations more resilient to extinction and with greater genetic diversity, and act as wildlife refugia (Roberts et al. 2017). To be effective, MPAs of any scale must be part of an environmental management package that includes improved land management for pollution, effective fisheries management, and reduced greenhouse gas emissions.

## Conclusions

Human activities have expanded across the oceans, and although intensity varies, few areas remain untouched (Halpern et al. 2015). With emerging activities such as deep-sea mining and marine biotechnology fast becoming reality, history suggests that exploitation pressures on the ocean will continue to increase and affected areas expand, rendering timely and increased protection vital (McCauley et al. 2015). Although LSMPAs are assets that already meet many immediate ecological and socioeconomic goals, their value will increase as the human footprint expands across the oceans. To achieve their full potential, however, MPAs of any size require effective implementation and management backed with strong protection (Edgar et al. 2014), and so it is essential that management ambition and protection level matches stated MPA objectives.

Clearly, no single strategy can protect marine biodiversity and resources. Polarized debates about the superiority of LSMPAs versus fishery or other management can divide a scientific and management community that shares the common goal of intelligently governing the future of the oceans for the benefit of humanity and all life within. Combining LSMPAs with effective management of all ocean uses, including fisheries, and other MPAs, such as smaller networked sites or dynamic MPAs, will establish a diversified management portfolio that tempers potential losses, insures against inherent ecological and management uncertainty, and ultimately enhances the probability of successfully achieving sustainably managed oceans.

## Supplementary Material

Supplemental data
