# Supplementary material for: Addressing Criticisms of Large-Scale Marine Protected Areas
Source: Bioscience. 2018 Apr 5;68(5):359–70. doi: 10.1093/biosci/biy021 (PMC5925769; doi:10.1093/biosci/biy021)
Supplement: Supplemental data [file biy021_supp.pdf]

**Addressing Criticisms of Large-Scale Marine Protected Areas - Supplementary Materials**

Bethan C. O'Leary, Natalie C. Ban, Miriam Fernandez, Alan M. Friedlander, Pablo García-Borboroglu, Yimnang Golbuu, Paolo Guidetti, Jean M. Harris, Julie P. Hawkins, Tim Langlois, Douglas J. McCauley, Ellen K. Pikitch, Robert H. Richmond, Callum M. Roberts

This file contains:

Table S1: Details of Large-Scale Marine Protected Areas as of January 2018

Methods (including Figure S1)

Threats from fisheries activities around LSMPAs (Figure S2)

**Table S1: Details of Large-Scale Marine Protected Areas as of January 2018 listed in reverse chronological order. LSMPAs that have been proposed but not yet legally designated are shown by an asterisk (*) next to the year. LSMPAs designated in overseas territories or remote waters falling under the jurisdiction of the designating country are indicated by a circumflex (^) next to country name. LSMPAs shown in shaded rows have been superseded (encompassed) by later LSMPA expansions. Where reserves have been expanded in later years the total area of the designation is given first followed by the additional area protected in the expansion in parentheses. Percentage no-take reported to nearest integer.**

| Year | LSMPA Name | Designating Country | Approx. surface area ($km^2$) | % fully/ strongly protected | Management Information and/or Plan Reference (listed in italics) |
|---|---|---|---|---|---|
| 2017* | Parque Nacional Revillagigedo | Mexico | 147,629 | 100 | Not yet legally designated. Announced at the Our Ocean 2017 conference (5-6th October, Malta) (available at: https://ourocean2017.org/our-ocean-commitments [accessed 06/10/2017]). Commercial fishing will be prohibited throughout. Justification for the LSMPA is available at: www.conanp.gob.mx/acciones/pdf/EPJ%20PN%20REVILLA%20-%207sept2017.pdf [accessed 16/11/2017]. |
| 2017* | Niue Marine Park | Niue | 127,000 | 100 | Not yet legally designated. Announced at the Our Ocean 2017 conference (5-6th October, Malta) (available at: https://ourocean2017.org/our-ocean-commitments [accessed 06/10/2017]). Commercial fishing will be prohibited throughout, traditional fishing will be permitted. |
| 2017* | Tallurutiup Imanga (Lancaster Sound National Marine Conservation Area) | Canada | 109,000 | Unknown | Not yet legally designated. Prior to establishment an Inuit Impact Benefit Agreement will be negotiated and agreed to decide on future management. No oil and gas exploration will be permitted. Details available at: www.cbc.ca/news/canada/north/lancaster-sound-marine-conservation-area-1.4246763 and www.iucn.org/news/protected-areas/201708/canada%E2%80%99s-newest-and-largest-marine-protected-area-tallurutiup-imanga-%E2%80%93-lancaster-sound [accessed 13/11/2017]. |
| 2017 | Marae Moana Marine Park | Cook Islands | 1,900,000 | 16 | Designated July 2017 through the Marae Moana Act 2017. Will be multiple use MPA with 16% in which no large scale commercial fishing or seabed minerals activities will be permitted, traditional fishing will be permitted. Plans announced at the UN Ocean Conference (June 5-9th 2017, New York) by the Cook Islands Government state the spatial plan will be delivered by 2020 (available at: https://oceanconference.un.org/commitments/?id=15701 [accessed 07/06/2017]). |
| 2017* | Juan Fernandez Archipelago MPA | Chile | 480,000 | Unknown | Not yet legally designated. Announced at the UN Ocean Conference (June 5-9th 2017, New York). Reaffirmed at Our Ocean 2017 (5-6th October, Malta). Will form part of the Cape Horn Biosphere Reserve. Details available at: https://oceanconference.un.org/commitments/?id=15763 and www.fis.com/fis/worldnews/worldnews.asp?l=e&country=0&special=&monthyear=&day=&id=92144&ndb=1&df=0 [accessed 13/06/2017]. |

| Year | LSMPA Name | Designating Country | Approx. surface area (km$^2$) | % fully/ strongly protected | Management Information and/or Plan Reference (listed in italics) |
|---|---|---|---|---|---|
| 2017* | Cabo de Hornos and Islas Diego Ramirez MPA | Chile | 100,000 | Unknown | Not yet legally designated. Announced at the UN Ocean Conference (June 5-9th 2017, New York). Reaffirmed at Our Ocean 2017 (5-6th October, Malta). Will form part of the Cape Horn Biosphere Reserve. Details available at: https://oceanconference.un.org/commitments/?id=15763 and www.fis.com/fis/worldnews/worldnews.asp?l=e&country=0&special=&monthyear=&day=&id=92144&ndb=1&df=0 [accessed 13/06/2017]. |
| 2017* | Te Tai Nui Atea (the Great Far Ocean) MPA | French Polynesia^ | 5,000,000 | Unknown | Not yet legally designated. Announced at the UN Ocean Conference (June 5-9th 2017, New York). Legislation framework, environmental and fisheries management plans intended to be adopted December 2017 with implementation of management plans by December 2020. Details available at: https://oceanconference.un.org/commitments/?id=20294 [accessed 13/06/2017]. |
| 2017 | Réserve naturelle nationale des Terres australes françaises (The French Southern Territories National Nature Reserve) | France^ | 1,662,766 (997,497) | 7 | Management plan for the period 2018-2027 is currently in preparation by the Scientific Council of the LSMPA (http://www.taaf.fr/Derniere-reunion-de-l-actuel-Conseil-scientifique-de-la-reserve-naturelle-nationale-des-Terres-australes-francaises-sur-l-elaboration-du-nouveau-plan-de-gestion-de-la-Reserve [accessed 04/10/2017]). The decree designating the LSMPA states that fishing may be conducted only when licensed by the State. Targeted shark and skate fisheries are prohibited as is the use of gillnets and bottom trawling. *Décret no 2016-1700 du 12 décembre 2016 portant extension et modification de la réglementation de la réserve naturelle nationale des Terres australes françaises*. Available at: www.taaf.fr/IMG/pdf/decret_extension_rnn.pdf [accessed 06/09/2017]. |
| 2016 | Réserve naturelle nationale des Terres australes françaises (The French Southern Territories National Nature Reserve) | France^ | 672,000 | 18 | Not applicable. LSMPA superseded by later designation |
| 2017 | Ross Sea MPA | High Seas | 1,550,000 | 72 | Designation came into force December 2017. *Ross Sea Region Marine Protected Area Management Plan* (Annex 91-05/B of CCAMLR Conservation Measure 91-05 (2016). Available at: https://www.ccamlr.org/sites/drupal.ccamlr.org/files//91-05_4.pdf [accessed 23/01/2017]. |
| 2016 | Galapagos Islands Marine Reserve (extension) | Ecuador^ | 178,000 (40,000) | 32 | *Dirección del Parque Nacional Galápagos. Plan de Manejo de las Áreas Protegidas de Galápagos para el Buen Vivir. 2014. Puerto Ayora, Galápagos, Ecuador* (available at: http://awsassets.panda.org/downloads/_1_plan_de_manejo_del_png_2014__final_.pdf [accessed 23/1/17]). |
| 2016* | Tristan da Cunha MPA | UK^ | 750,000 | Unknown | Not yet legally designated. Intended to be designated alongside a management plan by 2020. |

| Year | LSMPA Name | Designating Country | Approx. surface area ($km^2$) | % fully/ strongly protected | Management Information and/or Plan Reference (listed in italics) |
|---|---|---|---|---|---|
| 2016 | St Helena MPA | UK^ | 444,916 | 0 | St Helena Marine Management Plan (2016) prohibits commercial bottom trawling, tangle/gill nets, drift nets, purse seines, dynamite fishing, targeted shark or dolphin fishing, and fishing without a permit. Longlining must have 100% observer coverage. Available at: www.sainthelena.gov.sh/wp-content/uploads/2013/07/Marine-Management-Plan.pdf [accessed 04/10/2017]. |
| 2016* | Ascension Island Ocean Sanctuary | UK^ | 234,291 | 100 | Not yet legally designated. Intended to be designated alongside a management plan by 2019. |
| 2016 | Papahanaumokuakea Marine National Monument Expansion | US^ | 1,508,859 (1,146,798) | 100 | Established by 'Presidential Proclamation' (number not available) which prohibits all commercial and destructive practices and states that the existing management plan should be updated to include the Monument Expansion within 3 years of the proclamation (available at: https://obamawhitehouse.archives.gov/the-press-office/2016/08/26/presidential-proclamation-papahanaumokuakea-marine-national-monument [accessed 23/01/2017]). |
| 2016 | Nazca-Desventuradas Marine Park | Chile^ | 300,035 | 100 | The official decree designating the LSMPA states that management is to be developed by El Servicio Nacional de Pesca (SERNAPESCA) and the Servicion y la Subsecretaría de Pesca y Acuicultura (SUBPESCA) (available at: www.sernapesca.cl/index.php?option=com_remository&Itemid=246&func=fileinfo&id=20499 [accessed 04/10/2017]). Management plan (la gestión del Parque Marino Nazca-Desventuradas) is in preparation under the SUBPESCA project "Technical bases for the management of the Marine Park Nazca-Desventuradas and proposal of General Plan of Administration" (www.subpesca.cl/fipa/613/w3-article-96187.html and www.sernapesca.cl/index.php?option=com_content&view=article&id=2298:coordinan-esfuerzos-para-conservar-ecosistemas-marinos-en-islas-oceanicas-chilenas&catid=1:ultimas&Itemid=633 [accessed 04/10/2017]). |
| 2016 | Pitcairn Islands Marine Reserve | UK^ | 834,334 | 100 | No stand-alone management plan has been located. However, the *Pitcairn Islands MPA Ordinance 2016* provides much of the information regarding permitted activities and enforcement actions that would be expected from a management plan (available at: www.government.pn/Laws/2016-09-13%20Pitcairn%20Islands%20Marine%20Protected%20Area%20Ordinance%202016.pdf [accessed 23/01/2017]). Details on resources for management, monitoring and enforcement are not provided. |
| 2015 | Palau National Marine Sanctuary | Palau | 500,000 | 100 | *The Republic of Palau Exclusive Economic Zone Monitoring, Control, and Surveillance The next five years 2016-2021* (adopted by 'Presidential Directive 16-18') (available at: http://palaumarine.org/?page_id=45 under 'May 2016 Executive Committee Report' [accessed 23/01/2017]). Sanctuary to be fully established and in force by 2021. |

| Year | LSMPA Name | Designating Country | Approx. surface area (km$^2$) | % fully/ strongly protected | Management Information and/or Plan Reference (listed in italics) |
|---|---|---|---|---|---|
| 2015* | Rapa Nui Rahui MPA (previously Easter Island Marine Park) | Chile^ | 720,016 (569,676) | 88 | Not yet legally designated. Plans announced at the 4th International Marine Protected Area Congress (September 2017, Chile) by the President Michelle Bachelet state support from the Rapa Nui community for designation. Commercial fishing and other extractive/destructive activities will not be permitted throughout the LSMPA. A local fishing zone (c. 89,834 km$^2$) around Easter Island will permit the Rapa Nui to continue using the marine resources. No take area estimated from map published in The Guardian in 2015 (www.theguardian.com/environment/2015/sep/13/chile-plans-easter-island-marine-park-fishing [accessed 04/10/2017]). No further details available. Area of the LSMPA is based on the approximate size of the Easter Island EEZ (e.g. Zylich et al. 2014), although note that news reports are stating the LSMPA to be c.740,000 km$^2$. Once designated the Rapa Nui Rahui MPA would encompass the previously designated Motu Motiro Hiva Marine Park. |
| 2015* | Kermadec/Rangitāhua Ocean Sanctuary | New Zealand^ | 620,000 | 100 | Not yet legally designated. The Kermadec Ocean Sanctuary Bill is currently under review by New Zealand government (www.parliament.nz/en/pb/bills-and-laws/bills-proposed-laws/document/00DBHOH_BILL68514_1/kermadec-ocean-sanctuary-bill [accessed 04/10/2017]). If the Bill is passed a Conservation Board would be established to develop a management plan for the sanctuary although news reports as of September 2016 suggest the sanctuary designation has been delayed by ongoing negotiations with fishing operators who have existing property rights (www.radionz.co.nz/news/te-manu-korihi/313303/govt-to-delay-kermadec-ocean-sanctuary-bill). The government overview of the proposals states that it will be fully protected (www.mfe.govt.nz/marine/kermadec-ocean-sanctuary/about-sanctuary [accessed 04/10/2017]). |
| 2014 | Pacific Remote Islands Marine National Monument Expansion | US^ | 1,269,094 (1,054,125) | 100 | No management plan in place or in preparation. Reserve expanded by 'Presidential Proclamation 9173' which prohibits commercial fishing and states that a management plan should be developed within 2 years of the proclamation. *Compliance Guide Fishing in the Marianas Trench, Pacific Remote Islands, and Rose Atoll Marine National Monuments (revised April 2015)* states commercial fishing is prohibited (available at: http://www.fpir.noaa.gov/SFD/pdfs/monument-compliance-guide-Apr-2015.pdf [accessed 07/12/2017]). The Monument is managed by NOAA, the US Fish and Wildlife Service, and the Department of Defense (www.fpir.noaa.gov/MNM/mnm_prias.html [accessed 10/04/2017]. |

| Year | LSMPA Name | Designating Country | Approx. surface area (km$^2$) | % fully/ strongly protected | Management Information and/or Plan Reference (listed in italics) |
|---|---|---|---|---|---|
| 2014 | Le parc naturel de la mer de Corail (Natural Park of the Coral Sea) | France^ | 1,291,000 | Unknown | The Park's Management Plan (2017-2021) has been written and presented to the French government, (available at: https://mer-de-corail.gouv.nc/en/management-plan/parks-management-plan [accessed 04/10/2017]). Unable to locate a copy. The Department of Fisheries and Marine Environment of the New Caledonian Government is responsible for managing the LSMPA (www.affmar.gouv.nc/portal/page/portal/affmar/peche/presentation [accessed 10/04/2017]). According to MPAtlas the LSMPA will be multiple use with some no take areas. Spatial plans for the LSMPA are due by July 2018 (https://oceanconference.un.org/commitments/?id=19341 [accessed 04/10/2017]. |
| 2013 | Prince Edward Islands MPA | South Africa^ | 180,633 | 3 | *Prince Edward Islands Management Plan (version 0.2)* (available at: www.sanap.ac.za/sanap_ems/docs/marion_ems/PEIMP-Final%20web%20without%20sign/Prince%20Edward%20Islands%20Management%20Plan%20-%20PEIMP_v0_2.pdf [accessed 23/01/2017]). Management regulations of the National Environmental Management Protected Areas Act apply to the MPA (section 3.25). |
| 2012 | Charlie-Gibbs North High Seas MPA | High Seas | 178,651 | 0 | Designated by OSPAR who have no remit over fisheries. No management plan currently exists. Overlaps in part with NEAFC fisheries closure "Middle MAR" (currently in force till 31st December 2017, Rec 19 2014 www.neafc.org/system/files/Rec_19-2014_as_amended_by_09_2015_fulltext_0.pdf [accessed 04/10/2017]). |
| 2012 | Coral Sea Marine Park | Australia | 989,842 | 51 | Management suspended in 2013 pending review of the planning process. Consultation on draft management plans finished 20th September 2017 (https://parksaustralia.gov.au/marine/ [accessed 04/10/2017]). Plans announced at the UN Ocean Conference (June 5-9th 2017, New York) by the Australian Government state that Commonwealth Marine Reserves will be in force by December 2020 (https://oceanconference.un.org/commitments/?id=17908 [accessed 07/06/2017]). Renamed from a Commonwealth Marine Reserve to Marine Park in October 2017 (www.environment.gov.au/topics/marine/marine-reserves/overview/legal-framework [accessed 08/12/2017]). Strongly/fully protected area based on original zoning (http://www.environment.gov.au/topics/marine/marine-reserves/coral-sea/overview [accessed 04/10/2017]). |

| Year | LSMPA Name | Designating Country | Approx. surface area (km$^2$) | % fully/ strongly protected | Management Information and/or Plan Reference (listed in italics) |
|---|---|---|---|---|---|
| 2012 | South-west Corner Marine Park | Australia | 271,898 | 47 | Management suspended in 2013 pending review of the planning process. Consultation on draft management plans finished 20th September 2017 (https://parksaustralia.gov.au/marine/ [accessed 04/10/2017]). Plans announced at the UN Ocean Conference (June 5-9th 2017, New York) by the Australian Government state that Commonwealth Marine Reserves will be in force by December 2020 (https://oceanconference.un.org/commitments/?id=17908 [accessed 07/06/2017]). Renamed from a Commonwealth Marine Reserve to Marine Park in October 2017 (www.environment.gov.au/topics/marine/marine-reserves/overview/legal-framework [accessed 08/12/2017]). Strongly/fully protected area based on original zoning (http://www.environment.gov.au/topics/marine/marine-reserves/south-west/sw-corner [accessed 04/10/2017]). |
| 2012 | Norfolk Marine Park | Australia | 188,444 | 22 | Management suspended in 2013 pending review of the planning process. Consultation on draft management plans finished 20th September 2017 (https://parksaustralia.gov.au/marine/ [accessed 04/10/2017]). Plans announced at the UN Ocean Conference (June 5-9th 2017, New York) by the Australian Government state that Commonwealth Marine Reserves will be in force by December 2020 (https://oceanconference.un.org/commitments/?id=17908 [accessed 07/06/2017]). Renamed from a Commonwealth Marine Reserve to Marine Park in October 2017 (www.environment.gov.au/topics/marine/marine-reserves/overview/legal-framework [accessed 08/12/2017]). Strongly/fully protected area based on original zoning (http://www.environment.gov.au/topics/marine/marine-reserves/temperate-east/norfolk [accessed 04/10/2017]). |
| 2012 | Lord Howe Marine Park | Australia | 110,139 | 10 | Management suspended in 2013 pending review of the planning process. Consultation on draft management plans finished 20th September 2017 (https://parksaustralia.gov.au/marine/ [accessed 04/10/2017]). Plans announced at the UN Ocean Conference (June 5-9th 2017, New York) by the Australian Government state that Commonwealth Marine Reserves will be in force by December 2020 (https://oceanconference.un.org/commitments/?id=17908 [accessed 07/06/2017]). Renamed from a Commonwealth Marine Reserve to Marine Park in October 2017 (www.environment.gov.au/topics/marine/marine-reserves/overview/legal-framework [accessed 08/12/2017]). Strongly/fully protected area based on original zoning (http://www.environment.gov.au/topics/marine/marine-reserves/temperate-east/lord-howe [accessed 04/10/2017]). |

| Year | LSMPA Name | Designating Country | Approx. surface area (km²) | % fully/ strongly protected | Management Information and/or Plan Reference (listed in italics) |
|---|---|---|---|---|---|
| 2012 | Argo-Rowley Terrace Marine Park | Australia | 146,099 | 43 | Management suspended in 2013 pending review of the planning process. Consultation on draft management plans finished 20th September 2017 (https://parksaustralia.gov.au/marine/ [accessed 04/10/2017]). Plans announced at the UN Ocean Conference (June 5-9th 2017, New York) by the Australian Government state that Commonwealth Marine Reserves will be in force by December 2020 (https://oceanconference.un.org/commitments/?id=17908 [accessed 07/06/2017]). Renamed from a Commonwealth Marine Reserve to Marine Park in October 2017 (www.environment.gov.au/topics/marine/marine-reserves/overview/legal-framework [accessed 08/12/2017]). Strongly/fully protected area based on original zoning (http://www.environment.gov.au/topics/marine/marine-reserves/north-west/argo-rowley-terrace [accessed 04/10/2017]). |
| 2012 | South Georgia and South Sandwich Islands MPA | UK^ | 1,070,000 | 2 | *Government of South Georgia and the South Sandwich Islands (2013). South Georgia and the South Sandwich Islands Marine Protected Area Management plan* (available at: www.gov.gs/docsarchive/environment/#tab-2 [accessed 23/01/2017]). The first 5-year review of the LSMPA is currently being undertaken (www.gov.gs/environment/marine-protected-area/ [accessed 04/10/2017]). |
| 2010 | Motu Motiro Hiva Marine Park (also known as Salas y Gómez Marine Park) | Chile^ | 150,340 | 100 | Unable to locate details of management. The decree designating the LSMPA places it under the administration of the Servicio Nacional de Pesca and states that it will be governed by a management plan (www.subpesca.cl/portal/615/articles-2926_documento.pdf [accessed 04/10/2017].<br>Note. Should Easter Island Marine Park be designated, Motu Motiro Hiva Marine Park would be encompassed by this designation. |
| 2010 | Charlie-Gibbs South High Seas MPA | High Seas | 145,420 | 0 | Designated by OSPAR who have no remit over fisheries. No management plan currently exists. Overlaps in part with the North East Atlantic Fisheries Commission's fisheries closure "Middle MAR" (currently in force till 31st December 2017, Rec 19 2014 www.neafc.org/system/files/Rec_19-2014_as_amended_by_09_2015_fulltext_0.pdf [accessed 04/10/2017]). |
| 2010 | Chagos Marine Reserve | UK^ | 639,661 | 100 | *BIOT Interim Conservation Management Framework (2014)* (available at: http://biot.gov.io/wp-content/uploads/BIOT-Interim-Conservation-Management-Framework-September-2014.pdf [accessed 23/01/2017]. Interim because it recognises that UK and BIOT Government policies in the Territory may change with potential resettlement. Final plan to be developed. |
| 2009 | Pacific Remote Islands Marine National Monument | US^ | 214,969 | 100 | Not applicable. Reserve expanded in 2014. |

| Year | LSMPA Name | Designating Country | Approx. surface area (km$^2$) | % fully/ strongly protected | Management Information and/or Plan Reference (listed in italics) |
|---|---|---|---|---|---|
| 2009 | Marianas Trench Marine National Monument | US^ | 246,608 | 17 | Management plan in preparation and due to be released for public consultation early 2018 (Friedlander pers. comm.). Established by 'Presidential Proclamation 8335' which prohibited commercial fishing and states that a management plan should be developed within 2 years of the proclamation. *Compliance Guide Fishing in the Marianas Trench, Pacific Remote Islands, and Rose Atoll Marine National Monuments (revised July 2014)* states that commercial fishing is permitted in waters of the Trench and Volcanic Units but not in the Islands Unit of the Monument (available at: www.fpir.noaa.gov/SFD/pdfs/monument-fishing-compliance-guide_(rev.7-7-14).pdf [accessed 23/01/2017]). The Pew Charitable Trusts state that this is 16,405 square miles – this has been used to calculate no-take percentage (www.pewtrusts.org/en/projects/global-ocean-legacy-marianas [accessed 23/01/2017]). |
| 2008 | Phoenix Islands Protected Area | Kiribati | 408,250 | 99 | Unclear. Originally established with 3.1% no-take then expanded to 99.4% in 2015 with the remainder a restricted use zone. Draft management plan available but has expired with no replacement (*Phoenix Islands Protected Area Management Plan 2010-2014*). The *PIPA Climate Change Vulnerability Assessment and Management 2016* suggests management is in place and states the MPA is 99% no take. Both documents available from: www.phoenixislands.org/technical-publications.php [accessed 23/01/2017]. |
| 2006 | Papahanaumokuakea Marine National Monument | US^ | 362,061 (22,061) | 100 | Not applicable. Reserve expanded in 2016. |
| 2000 | Northwestern Hawaiian Islands Coral Reef Ecosystem Reserve | US^ | 340,000 | Unknown | Not applicable. LSMPA superseded by Papahanaumokuakea Marine National Monument in 2006. |
| 1999 | Macquarie Island Marine Park | Australia | 162,000 | 36 | Proclaimed in 1999 as the Macquarie Island Marine Park, renamed in 2007. Managed under the *South-east Commonwealth Marine Reserves Network Management Plan 2013-23* (available at: www.environment.gov.au [accessed 23/01/2017]). Renamed from a Commonwealth Marine Reserve to Marine Park in October 2017 (www.environment.gov.au/topics/marine/marine-reserves/overview/legal-framework [accessed 08/12/2017]). |
| 1998 | Galapagos Islands Marine Reserve | Ecuador^ | 138,000 | 0 | Not applicable. Reserve expanded in 2016. |
| 1975 | Great Barrier Reef Marine Park | Australia | 344,000 | 33 | Management has been subdivided into four management areas: Cairns Area, Hinchinbrook, Shoalwater Bay, and Whitsundays. Management plans for each can be accessed from: www.gbrmpa.gov.au [accessed 23/01/2017]. |

**Methods**

Median global MPA size and global protected area coverage statistics were calculated using the January 2017 version of the World Database on Protected Areas (WDPA) (IUCN and UNEP-WCMC 2017) updated with information gathered by the authors on LSMPAs. LSMPA no-take coverage statistics were based on information described in Table S1. Where the no-take coverage was unknown it was assumed to be zero in these calculations and for presentation purposes in Figure 2. A total of 13,075 MPAs were included in analyses. Note that while we have excluded single-species and fishery management areas from our definition of a LSMPA, this database includes these areas and is therefore likely a generous estimate of global MPA coverage. Having said that, the WDPA database appears to have incomplete records for 2015 and 2016 based on the number of records in the database (Figure S1), and so care is needed when interpreting these figures. Only nine records were included in 2017, the LSMPAs 'Réserve naturelle nationale des Terres australes françaises' (France), Te Tai Nui Atea (the Great Far Ocean) MPA (France), Juan Fernandez Archipelago MPA (Chile), Cabo de Hornos and Islas Diego Ramirez MPA (Chile), Marae Moana Marine Park (Cook Islands), Niue Marine Park (Niue), Tallurutiup Imanga (Lancaster Sound National Marine Conservation Area, Canada), Revillagigedo Marine Reserve (Mexico), and Ross Sea MPA (high seas) added by the authors.

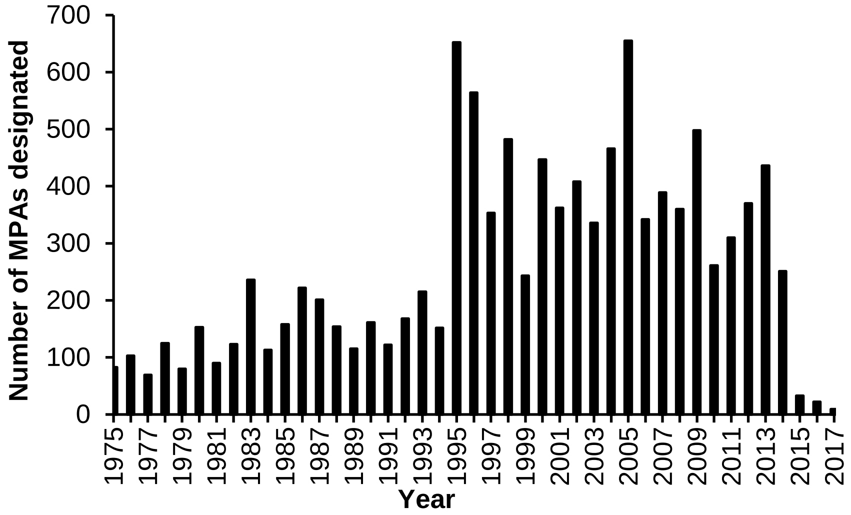


**Figure S1**: Number of MPAs designated each year globally since 1975 according to the WDPA database as of January 2017 and author LSMPA additions as of January 2018.

For consistency with our definition, and to ensure large-scale (≥100,000 $km^2$) single-species and fishery management areas did not inflate estimates of global coverage, these were removed from the database. A total of 1,144 MPAs were excluded: 510 UNESCO Man and the Biosphere Reserves (MAB reserves) and Ramsar sites (wetlands of international importance) in line with other global studies of MPAs (e.g. Boonzaier and Pauly 2016, UNEP-WCMC and IUCN 2016, Wood et al. 2008); 21 MPAs with a status of "not-reported"; 604 MPAs reported without an associated area; and 9 single-species and fishery management areas with an area ≥100,000 $km^2$. Note that these are listed in the order of removal and therefore do not represent absolute numbers in each category. An additional 21 LSMPAs were added to the database. MPAs with a status of "proposed" were included in analyses for consistency with our inclusion of LSMPA designations.

Total MPA coverage was calculated after removal of all overlapping areas using the Dissolve tool in ArcGIS 10.3.1 and using the Cylindrical Equal Area projection. Finally, the cumulative area of MPAs

designated globally over time is only indicative of trends in global MPA coverage (Figure 1b) and was based on the January 2017 version of the WDPA (IUCN and UNEP-WCMC 2017) following the World Database on Protected Areas User Manual 1.4 guidelines. Note that MPAs where the year of designation was not known were removed and areas of overlap remain in this analysis.

Finally, it is important to note that there is some disagreement as to the actual area protected in implemented and actively managed MPAs around the world. Analysis by the Atlas of Marine Protection (www.MPAtlas.org) suggests that only 3.6% of the world's oceans are in MPAs compared with the 6.6% suggested by the WDPA as of January 2017. This distinct is not trivial and suggests that the WDPA overestimates the level of protection being provided to marine ecosystems.

**Threats from fisheries activities around LSMPAs**

Figure S2 shows the global distribution of commercial fishing. The low overlap between designated and promised LSMPAs and commercial fishing activities suggests that displacement of fishers is not currently a major concern of LSMPAs.

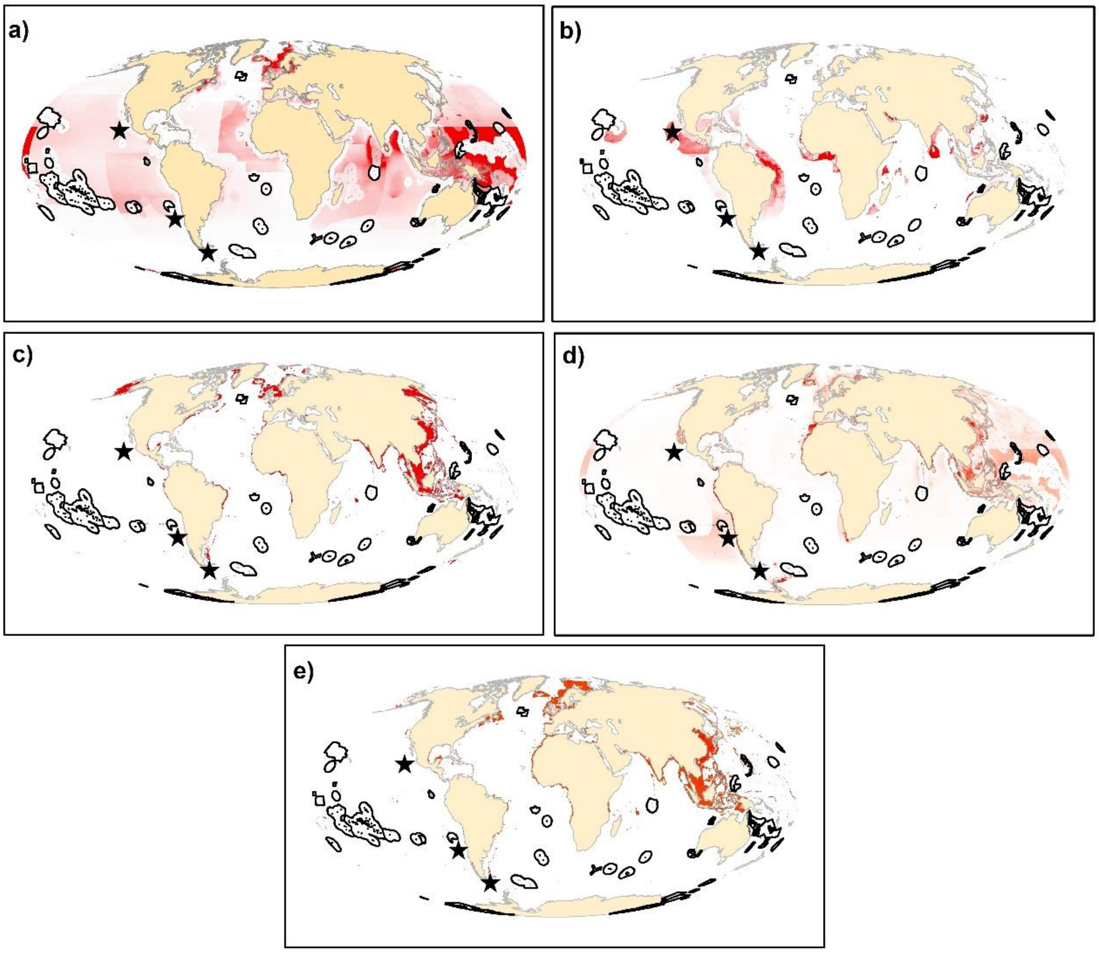


**Figure S2:** Global distribution of commercial fisheries activity for (a) pelagic low-bycatch fishing, (b) pelagic high-bycatch fishing, (c) demersal destructive fishing, (d) demersal non-destructive low-bycatch fishing, and (e) demersal non-destructive high-bycatch fishing. White areas indicate no activity or no data, red areas indicate places subject to higher intensity of use. Data freely available from Halpern et al. (2015, 2008).

**References**

Boonzaier L, Pauly D. 2016. Marine protection targets: an updated assessment of global progress. Oryx 50:27-35.
Halpern BS, et al. 2015. Spatial and temporal changes in cumulative human impacts on the world's oceans. Nature Communications 6:7615.
Halpern BS, et al. 2008. A Global Map of Human Impact on Marine Ecosystems. Science 319:948-952.
IUCN, UNEP-WCMC. 2017. The World Database on Protected Areas (WDPA) [On-line], [Jan 2017]. (24/01/2017 www.protectedplanet.net)
UNEP-WCMC, IUCN. 2016. Protected Planet Report 2016. UNEP-WCMC and IUCN: Cambridge UK and Gland, Switzerland. Report no.
Wood LJ, LFish L, Laughren J, Pauly D. 2008. Assessing progress towards global marine protection targets: Shortfalls in information and action. Oryx 42:1-12.
Zylich K, Harper S, Licandeo R, Vega R, Zeller D, Pauly D. 2014. Fishing in Easter Island, a recent history (1950 2010). Latin American Journal of Aquatic Research 42:845-856.
